# Embracing Letters to the Editor: Classifying Types of Letters into Disagreement, Agreement, and Complementary

**DOI:** 10.31662/jmaj.2024-0053

**Published:** 2024-08-09

**Authors:** Shigeki Matsubara

**Affiliations:** 1 Department of Obstetrics and Gynecology, Jichi Medical University, Tochigi, Japan; 2 Department of Obstetrics and Gynecology, Koga Red Cross Hospital, Koga, Japan; 3 Medical Examination Center, Ibaraki Western Medical Center, Chikusei, Japan

**Keywords:** categorization, classification, correspondence, letter, manuscript

## Abstract

“Letters to the Editor” that address original articles significantly contribute to medical literature. Utilizing letters published in obstetrics and gynecology journals as a study model, I aimed to classify letters according to their context, providing a valuable framework for readers to comprehend the significance of letters and for authors to effectively write them. Using a sample of 40 recent letters from the Journal of Obstetrics and Gynaecology Research (JOGR), I classified letters into three main categories based on their attitude to addressed articles: Disagreement, Agreement, and Complementary. I further subclassified each category into subcategories, including “Interpretation claim,” “Data addition,” and “Historical viewpoint.” The same procedure was carried out for the 24 most recent letters from BJOG and the American Journal of Obstetrics and Gynecology and also for the JMA Journal. Disagreement letters were prevalent in all three OBGYN journals, accounting for 1/2 to 2/3 of all letters. The rest letters were categorized either as “Agreement” or “Complimentary.” Subcategorizations demonstrated different ratios of letters of the three categories among journals. I believe that the attempt to categorize and subcategorize letters offers valuable insights into the letters, potentially enhancing clarity in medical literature communication.

## An Attempt to Classify Letters

Exchanging opinions is crucial in medical progress. In the medical publication, a letter to the editor plays a pivotal role in this exchange. A Letter, providing a platform for changing opinions, often augments the overall value of the addressed article.

I recognize that there are letters with different contexts; however, to my knowledge, no attempts have been made to classify letters based on their contexts. I believe that such classifications of letters may help readers and authors grasp the letter’s context, thereby helping them read and write letters.

My major is obstetrics and gynecology (OBGYN). I have written and reviewed several manuscripts for the Journal of Obstetrics and Gynaecology Research (JOGR), a formal journal of the Japan Society of Obstetrics and Gynecology. I read almost all JOGR articles at a new issue release. I can analyze OBGYN articles, especially those from JOGR, more adequately than those from other specialties. Therefore, I first aimed to work on JOGR Letters. I also analyzed those from two other leading OBGYN journals and touched on those from the JMA Journal. I worked on classifying letters based on their context, providing a valuable framework for readers to comprehend the significance of letters and for authors to write them effectively.

## Retrieving Letters from JOGR and Classifying and Subclassifying Them

On March 5, 2024, I retrieved letters published in JOGR. Using a PubMed search with the query “J Obstet Gynaecol Res letter,” I identified 221 articles, encompassing various types including letters that address published articles, independent letters, and replies. The targets were the first type (referred to as “Letter(s)”). I examined all Letters published in JOGR from the 2018 March Issue to the 2024 March Issue, covering 6 years. The total number was 40.

I carefully read these 40 Letters and the addressed articles. Based on the Letters’ attitude toward addressed articles, I classified them into three categories: “Disagreement,” “Agreement,” and “Complementary” Letters. I defined them as follows:

1) Disagreement Letters point out some weaknesses of the addressed articles.

2) Agreement Letters add some other data, supporting the context of the addressed articles.

3) Complementary Letters, regardless of disagreement or agreement, demonstrate aspects other than those presented in the addressed articles.

Some Letters have several aspects of the three categories (i.e., one Letter has aspects of Disagreement and Complementary). In this case, I considered which aspect was the main context and occupied the space most in the Letter. In a more detailed manner, many Letters begin with a remark, such as “I commend the authors for providing valuable data,” which does not always “agree” with the context of the original article. Thus, I carefully determined the main context of the Letter. Subsequently, I subclassified each type of Letter. For the Disagreement Letters, I subclassified them based on the fundamentals of paper writing ^(1)^. For the remaining two, I subclassified them according to the Letter’s context.

## Three Categorizations of Letters

**Figure 1.**
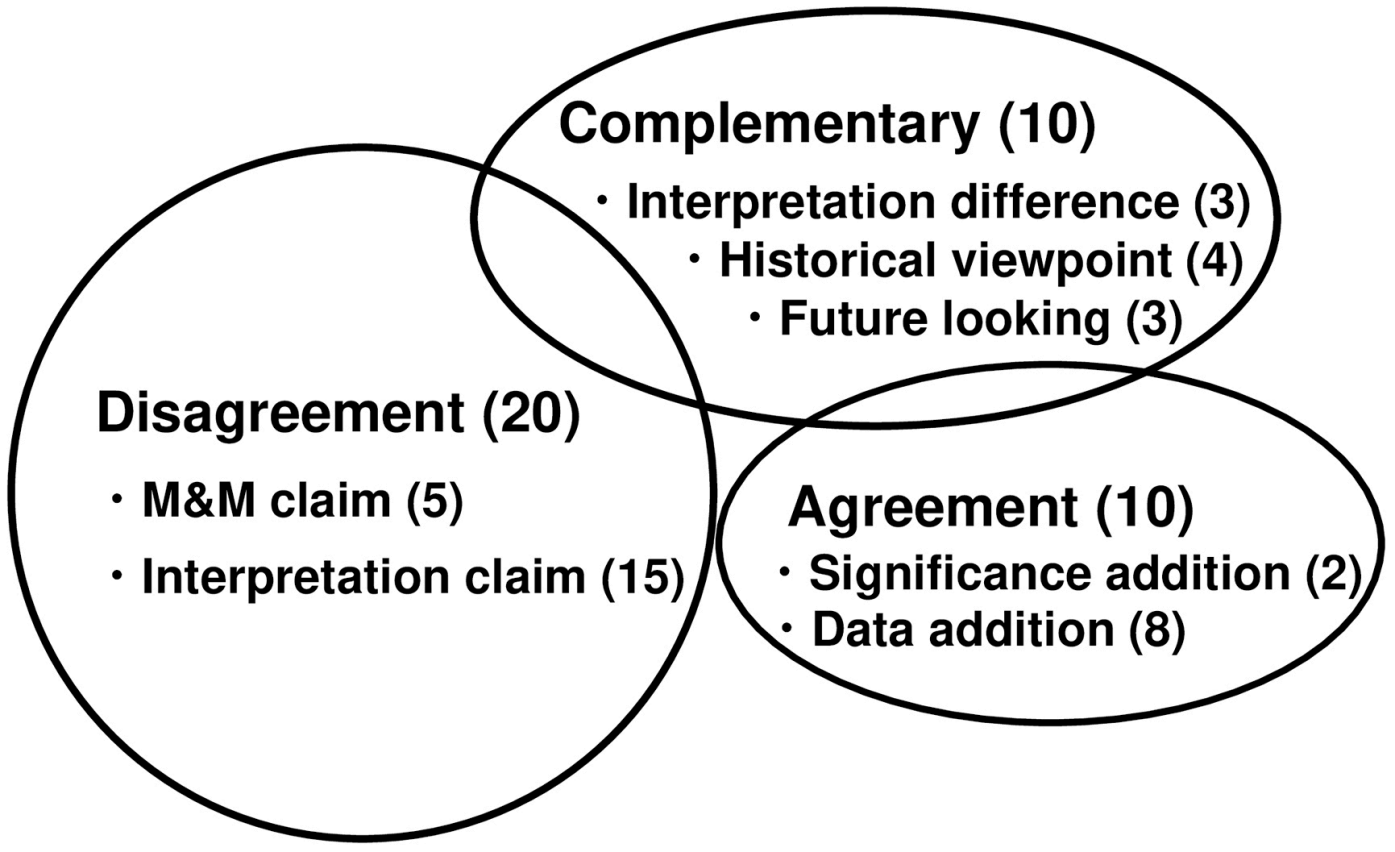
Schematic presentation of three classifications of letter to the editor (Letter). Letters can be classified into three categories, Disagreement, Agreement, and Complementary (see text). Some Letters contain overlapping contexts, accounting for circles’ overlapping. In each category, some subcategories exist according to the context of the Letter. The number in parenthesis indicates that of Letters most recently published in the Journal of Obstetrics and Gynaecology Research.

Of the 40 JOGR Letters, 20, 10, and 10 were classified as Disagreement, Agreement, and Complementary, respectively (Figure 1). Disagreement Letters criticize various parts of the original manuscripts corresponding to the fundamentals of paper writing (paper structure) ^(1)^.

1. Disagreement Letters (n = 20, 20/40, 50%) address the Materials and Methods (M&M), Results, and Discussion sections of the original article. The introduction usually comprises three parts: known, unknown, and the present problem ^(1)^. Disagreement with the Introduction implies that the present problem is deemed unworthy of study. No Letters were found challenging the Introduction, indicating that none questioned the study’s worthiness. Five Letters claimed the M&M (M&M claim). Selection bias and wrong statistics principally account for this type. Fifteen Letters claimed incorrect data analysis or interpretation, the Results and Discussion section (Interpretation claim) (Table 1, Example 1).

**Table 1.** Examples of Letter Categorizations and Subcategorizations.

**Disagreement**
**Example 1 (Interpretation claim):** The original article compared the number of OBGYN papers in a country-by-country manner and thereby ranked countries. The Letter claimed that other factors including gross national products or the country’s population should be considered.☆
**Agreement**
**Example 2 (Significance addition):** An original article showed the efficacy and safety of ferric carboxymaltose for the treatment of anemic pregnant women. The Letter claimed that the vital parameters demonstrated here will detect hypersensitive reactions during iron infusion.
**Example 3 (Data addition):** An original article showed surgical repair of non-scarred uterine rupture during pregnancy. The Letter added a similar experience, which supports this strategy.
**Complementary**
**Example 4 (Interpretation difference):** The original article showed a case of asymptomatic uterine rupture. The Letter interprets such cases under the concept of “masked uterine rupture.”☆☆
**Example 5 (Historical viewpoint):** The original article showed a new procedure of uterine compression suture in which the thread is removed after hemostasis. The Letter summarizes the research history of “removable” compression sutures.
**Example 6 (Future looking):** The original article demonstrated that “cold” relieved mothers’ pain during the amniocentesis procedure. The Letter proposed to study the cold effect on the fetal side.
**Example 7 (Complementary, Interpretation difference, JMA Journal):** The original article claimed the importance of educating trainees to write case reports. The Letter suggested that senior doctors should write case reports depending on the situation, a complementary viewpoint.

☆versus ☆☆: Recognize the difference between a Disagreement Letter (Interpretation claim; Example 1)^☆^ and a Complementary Letter (Interpretation difference; Example 4).^☆☆^ With both focusing on interpretation, the former criticizes the original article, whereas the latter provides a different interpretation without criticism. Examples 1-7 are from the following reference, with [number] indicating each example shown above. [1] J Obstet Gynaecol Res (JOGR) 2019;45:1085, [2] JOGR 2022;48:515, [3] JOGR 2020;46: 2702, [4] JOGR 2020;46:1927, [5] JOGR 2018;44:2016, [6] JOGR 2021;47:3742, [7] JMA J. 2023;14;6(3):362.

2. Agreement Letters (n =10, 10/40, 25%): Expecting that one agrees with some specific parts of the Introduction, M&M, Results, or Discussion is difficult. Thus, I adopt viewpoints different from those used for Disagreement Letters. Two Letters added “significance” to the original article (Significance addition) and eight added data from the authors of the Letters or preexisting data (Data addition), thereby both supporting the original article’s context (Table 1, Examples 2 and 3, respectively).

3. Complementary Letters (n = 10, 10/40, 25%): This type is classified into three: showing that the data can be interpreted differently (Interpretation difference) (n = 3), touching historical perspective (Historical viewpoint) (n = 4), and showing future study direction (Future looking) (n = 3) (Table 1; Examples 4, 5, and 6, respectively).

## Letters Published in Some Journals Other than JOGR

On March 6, 2024, I examined BJOG and the American Journal of Obstetrics and Gynecology (AJOG). BJOG is the official journal of the Royal College of Obstetricians and Gynaecologists in the United Kingdom. AJOG, a journal based in the United States, holds the highest impact factor among OBGYN category journals worldwide and publishes a substantial number of Letters. For BJOG, PubMed with “BJOG and letter” retrieved the most recent 24 Letters, with early view dates June 26, 2023, to March 4, 2024, covering 8 months. For AJOG, checking all Letters from Volume 229, Issue 5 (November) 2023 to Volume 230, Issue 3 (March) 2024 (covering 5 months) yielded 24 Letters. I classified and subclassified these 24 Letters from BJOG and AJOG. I retrieved and analyzed all Letters from the JMA Journal, Volume 1, Issue 1, 2018 to Volume 7, Issue 1, 2024 (covering 6 years).

Letters published in BJOG and AJOG (both; n = 24) are as follows: Disagreement (BJOG vs. AJOG: 13 vs. 16) (M&M claim, 7 vs. 11; Interpretation claim, 6 vs. 5), Agreement (9 vs. 2) (Significance addition, 1 vs. 0; Data addition, 8 vs. 2), and Complementary (2 vs. 6) (Interpretation difference, 0 vs 0; Historical viewpoint, 1 vs. 2; Future looking, 1 vs. 4). Letters from the JMA Journal numbered 6: Disagreement (2; Interpretation claim, 2), Agreement (3; Data addition, 3), and Complementary (1; Interpretation difference, 1 (Table 1, Example 7)).

## A Balanced Publication of Three Categories

Disagreement vs. Agreement vs. Complementary Letters published in JOGR were 50% vs. 25% vs. 25%, respectively. It may be feasible to say that Disagreement Letters outweigh Agreement Ones because Letters of the latter style cannot stand alone in academic journals. This research clarified an important aspect about various ratios of Letters of the different three categories between journals, although the ideal “ratio” is still unknown.

## Meaning of Complementary Letters

Describing Complementary Letters may require deep, wide, and historical viewpoints. I never intend to imply that Letters of a certain category are superior to others. If a Letter is useful to the readers, it is a good Letter, regardless of the category. However, generally speaking, compared with Letters indicating the presence of “statistics wrong,” Complementary Letters often introduce new insights that the original paper did not address. Complementary Letters are principally more likely to touch on the issue more generally. I believe that we must embrace such a Letter.

## Analyses Including Other Journals: Data Generalizable

BJOG and AJOG also published Letters of all three categories and Disagreement Letters dominated as well (13/24 and 16/24, respectively). Different journals (JOGR vs. BJOG vs. AJOG) had different percentages among categories and subcategories. Each journal may have different policies for publishing Letters. I do not delve into a comparison of Letters’ types among these journals because I do not know the journal policies, and I also cannot imagine that such a discussion would lead to information useful for readers. Rather, notably, Letters of all three categories are published in all three journals, irrespective of their geographic origin. Moreover, Letters from all three journals belonged to some subcategories. Thus, the present categorizations and subcategorizations are considered not specific to JOGR Letters. Letters published in the JMA Journal, although their number is small, also belonged to some of the three categories and subcategories.

## Merits of the Letter

How do Letters merit readers of the journal and authors of the addressed articles? With the following two examples, I want to insist that one merit may be “enriching discussions with resonation of authors through Letters.” In Example 5, the original article ^(2)^ described a new “removable” uterine compression suture, without mentioning some other similar procedures previously described. The Letter (Example 5) pointed it out and summarized removable suture hitherto reported. Then, the authors of the original article replied, adding some other removable suture techniques ^(3)^. This good communication may be due to the author’s perception, “This letter aims to enrich the original article by adding a historical viewpoint, rather than criticizing its omission of previous articles.” In the JMA journal, I penned a Letter, claiming that case reports should not be “exclusive possession” of trainees (Example 7). The author of the addressed article replied and provided a new point: Continuously writing Letters and participating in review processes may encourage trainees ^(4)^. In both cases, Letters with a straightforward context resonated with the authors of the articles addressed, prompting them to write meaningful Replies. Both Letters and Replies merited all; the article’s authors, the Letter’s authors, and readers. This enhanced academic communication among all.

Although very experienced readers and writers can do it without such classifications, for those with less experience, grasping the categories (Disagreement, Agreement, or Complementary) and subcategories may help readers better follow the context and also help authors write things clearly and consistently.

## Significance and Limitation

I focused on Letters from OBGYN journals in this article. Nevertheless, I believe that the classification gained here can be generalizable to other specialties because my study treated general aspects of academic articles. The following limitation should be declared: The number of Letters examined was relatively small, and no double-check was performed by another individual besides myself. Criteria of classification and subclassification were subjective. A real-world usefulness has not been shown.

## Embracing Letters

I love to read and write Letters, a vivid human voice. In this article, I aimed to classify Letters’ types to clarify and enhance their academic significance. Through this article, I hope that readers, having read up to this point, come to love Letters more by realizing their academic importance.

## Article Information

### Conflicts of Interest

None

### Acknowledgement

I thank Eiko Matsubara for her secretarial assistance.

### Author Contributions

S. Matsubara: Identification of the significance. Manuscript writing.

### Approval by Institutional Review Board (IRB)

Not applicable

### Patient Anonymity

Not applicable

### Informed Consent

Not applicable

### Data Availability Statement

The data that support the findings of this study are available in PubMed and home page of the American Journal of Obstetrics and Gynecology [https://www.ajog.org] and of JMA Journal [https://www.jmaj.jp/index.php] (Accessed 30 March 2024).

## Supplement

A Supplementary File PubMed ID (PMID) numbers for 40 Letters published in the Journal of Obstetrics and Gynaecology Research, classified and subclassified into categories and subcategories.
