# Supplementary material for: Embracing Letters to the Editor: Classifying Types of Letters into Disagreement, Agreement, and Complementary
Source: JMA J. 2024 Aug 9;7(4):610–4. doi: 10.31662/jmaj.2024-0053 (PMC11543308; doi:10.31662/jmaj.2024-0053)
Supplement: A Supplementary File — PubMed ID (PMID) numbers for 40 Letters published in the Journal of Obstetrics and Gynaecology Research, classified and subclassified into categories and subcategories. [file 2433-3298-7-4-0610-s001.pdf]

A supplementary file

PubMed ID (PMID) numbers for 40 Letters published in the Journal of Obstetrics and Gynaecology Research, classified and sub-classified into categories and sub-categories.

Star (☆) indicates Letters described in Table 1. For example, ☆2 indicates the Letter of "Example 2".

1. Disagreement
   M&M claim: 37088777, 35142411, 30723970, 30575233, 29171102
   Interpretation claim: 34558149, 34525487, 34365678, 33969581, 33269516, 32648997, 32343040, 30811769[☆1], 30393910, 30288864, 29974614, 29932489, 29744973, 29644779, 29607576
2. Agreement
   Significance addition: 34806819[☆2], 29673004
   Data addition: 35274402, 34494342, 33006266[☆3], 32985038, 30251427, 29442410, 29359481, 29323462
3. Complementary
   Interpretation difference: 36935191, 36859616, 32643175[☆4]
   Historical viewpoint: 34155727, 33870600, 30015366[☆5], 29570918
   Future looking: 37309742, 34263503[☆6], 30276932
